# Functional Management of Waste Wood Flour as an Example of a ‘Greener’ Approach towards the Synthesis of Bio-Based Epoxy Resins

**DOI:** 10.3390/polym15173521

**Published:** 2023-08-23

**Authors:** Anna Sienkiewicz, Piotr Czub

**Affiliations:** Department of Chemistry and Technology of Polymers, Faculty of Chemical Engineering and Technology, Cracow University of Technology, Warszawska Str. 24, 31-155 Kraków, Poland; piotr.czub@pk.edu.pl

**Keywords:** epoxy resin, modified soybean oil, wood/polymer composite, vegetable oil modification, oak wood flour waste, epoxy fusion process

## Abstract

Nowadays, in the era of growing ecological awareness, composites based on synthetic or bio-based polymers and fillers of natural origin find various potential applications. Plant-based materials are obtained using plant-derived materials, such as e.g., vegetable oil or wood fillers. Such synthesis of polymer composites allows for the selection of the reactants in terms of the potential requirements of the application. In the presented research polymer composites were obtained using bio-based high molecular-weight epoxy resins of hydroxylated soybean oil (SMEG) and a low-molecular-weight epoxy resin (EPR 0162) filled with the oak wood flour waste from the production of parquet flooring. To increase the poor compatibility between the highly hydrophilic wood fibers and the hydrophobic polymer matrix, waste wood flour (WF) was subjected to chemical modifications (mercerization, acetylation, and diisocyanate modification). Based on performed FT-IR and SEM analysis of wood flour, it was found that, among all performed modifications, the acetylation allows for the hydroxyl groups removal to the greatest extent. As a result of sequence synthesis including (1) the synthesis of SMEG_EPR polyaddition product, (2) the introduction of WF followed by its (3) curing with diisocyanate, obtained wood/polymer composites contain about 40% of raw materials of natural origin. As a consequence of the carried out modification of the wood waste flour, the compatibility of the filler and the bio-based polymer matrix was improved, resulting in an improvement in compressive strength by 3.51 MPa (SMEG_EPR_2% WF-10% NaOH) and 2.19 MPa (SMEG_EPR_2% A-WF) compared to samples containing unmodified wood flour. Additionally, concerning the results registered for pure SMEG_EPR composition, the introduction of 2 wt.% of wood filler resulted in a three/fourfold increase in the elongation at the break of the composition containing unmodified and chemically modified wood flour (10.99%—SMEG_EPR_2%WF; SMEG_EPR_2%WF-5%NaOH–10.36%; SMEG_EPR_2%WF-10%NaOH–9.54%, and 12.15%—SMEG_EPR_2%A-WF). Moreover, the incorporation of wood filler increased the value of the compression set of samples (2.40%—SMEG_EPR_2%WF, 2.39%—SMEG_EPR_2%WF-5%NaOH, and 2.34% for SMEG_EPR_2%WF-10%NaOH compared with 2.32%—SMEG_EPR).

## 1. Introduction

As of 2019, the global production of plastics and plastic products exceeded 368 million tonnes and was 2.5% higher than in the previous year. However, in the first half of 2020, as a result of pandemic constraints, a sharp decline in plastic production was observed. Then polymer production began to increase again in the second half of 2020. The recovery of the plastics industry in the European Union is expected to continue, but pre-pandemic polymer production will not be achieved until the end of 2022 [1]. Nevertheless, the constantly growing production of polymer materials is a direct cause of the increase in the consumption of petrochemical raw materials, and thus-their prices. Research is underway on the acquisition of new materials of natural origin that can be successfully used in the production of polymeric materials. Studies on novel materials based on epoxy resins are conducted in this trend as well [2,3,4].

Epoxy resins are one of the most important polymeric materials, which for several decades have been the main subject of many patents, as well as scientific and technical solutions. Although they have been used on an industrial scale since the 1940s [5], they are still successfully used in the synthesis of modern materials. Cured epoxy resins are characterized by exceptional physicochemical properties, such as good mechanical strength, thermal stability, and polarity [6]. Epoxy resins are used as protective layers of building structures, coatings for floors and furniture, powder paints, and, thanks to their electrical insulating properties, for cable connections of electrical installations [7,8]. Epoxy resins are also perfect as materials resistant to unfavorable weather conditions, characterized by good mechanical strength and relatively low weight. Therefore, they are also widely used in the demanding aviation industry as structural elements of aircraft or wind turbines [9]. 

Taking into account the ecological and social considerations attempts have been made to obtain epoxy materials based on e.g., vegetable oils. The most commonly used natural oils used for this purpose include castor oil, rapeseed oil, soybean oil, palm oil, peanut oil, linseed oil or cotton oil [10,11,12,13]. However, they must first be modified by e.g., epoxidation or opening of oxirane rings in epoxidized oils [14,15]. The most commonly used and described in the literature methods for vegetable oil oxidation are epoxidation via the Prilezhaev process, radical oxidation, Wacker-type oxidation, dihydroxylation of oils and fats, and epoxidation using heterogenous catalysts such as enzyme oxidation [16,17], HY zeolite, sulfated-SnO_2_, Nb–SiO_2_, CoCuAl layered double hydroxides, Ti-incorporated mesoporous silica and N_2_O_5_–SiO_2_ [18]. Modified vegetable oils can be used as stabilizers and plasticizers for thermoplastics. They can also be used in the synthesis of high-molecular epoxy resins [19,20,21] and replace polyols of petrochemical origin in the reactions of obtaining polyurethane materials [22,23]. Over the past few years, in our research group, we have performed studies on the application of modified vegetable oil as raw materials in the synthesis of high-molecular-weight resins conducted by the polyaddition reaction carried out in bulk via the epoxy fusion process. In our opinion, this pro-ecological method is simple and allows us to obtain products with a pre-determined molecular weight, much higher than during the conventional solvent method. The products are in the form of liquid but are characterized by higher resilience. Additionally, what is especially important from the ecological point of view, the epoxy fusion process allows for the partial replacement of petrochemical resources by modified vegetable oils, as well as eliminating harmful environment solvents, strong acids and alkali. 

On the other hand, due to the growing interest in biodegradable materials and recyclable products, numerous studies have also been carried out on the use of various types of fillers and natural reinforcements to lower the cost of polymer composites. There are numerous studies on the application of various natural-based fillers for polymer composites, such as rice husk [24], corn husk [25], peanut shell [26], coconut shell [27], sorghum [15], etc. Even though the use of wood-based fillers is not as popular as the use of mineral or inorganic fillers, wood-derived fillers are characterized by several advantages, such as low density, flexibility during the processing with no harm to the equipment, acceptable specific strength properties, and low cost per volume basis [28]. Well-known wood fillers are in the form of powders, flour, or fibers. The introduction of natural fibers into a polymer matrix can result in improved mechanical and thermal properties. However, the problem with the introduction of natural fibers into composites is the relatively low adhesion between the fiber and the polymer material, as well as the relatively high moisture sorption by the natural filler. Wood fillers consist of cellulose (approx. 50%), hemicelluloses (approx. 20%), such as xylans, manganese, acetic esterase, lignin (approx. 26%) and other substances, such as poliosis (similar in structure to hemicelluloses) and tannins (condensed polyphenols) [29]. Wood hygroscopicity related to the adsorption and desorption of water vapor from the environment results from the presence of cellulose and hemicelluloses in the structure of the wood. Since water vapor particles are bipolar, they have electrically positive and negative poles. Negatively charged -OH groups in cellulose, absorbed water molecules, which force them to a specific orientation leading to the spacing of the surface micelles. Increasing the distance between the micelles weakens the forces of cohesion between them, and thus the mechanical properties of wood deteriorate. Wood flour is often modified chemically, physically, and biologically to increase the strength of the particles, decrease water absorption, or improve composite properties generally. The most frequently performed chemical modifications are mercerization [30], acetylation [31,32], silanization [33], treatment with permanganate potassium [34], or organic peroxides [35]. All these reactions lead to the substitution of the hydroxyl groups of the cellulose by less polar groups, which decreases water adsorption and the tendency for aggregation.

This study aims to determine the effect of wood flour in wood/polymer composites based on high molecular weight epoxies from hydroxylated soybean oil and bisphenol A-based epoxy resin. The scope of the undertaken research included the modification of soybean oil towards the synthesis of the hydroxylated derivative and obtaining high-molecular epoxy resin in a polyaddition reaction with a low molecular weight epoxy resin. Finally, developing methods and conditions for modifying wood flour to improve the compatibility of the filler with the polymer matrix and the analysis of selected mechanical properties of the obtained epoxy-polyurethane composites filled with wood flour. Simultaneously, undertaken research is an attempt at functional management of waste wood flour as an example of a ‘greener’ approach towards the synthesis of bio-based epoxy resins.

## 2. Materials and Methods

Materials. Epoxidized soybean oil (ESBO, Ergoplast EG, Boryszew, Poland, EV = 0.363 mol/100 g; with an average of 3.52 epoxide groups, and 0.03 hydroxyl groups per 1 oil molecule; both evaluated by titration methods according to standards described in our previous paper [36]), ethylene glycol (EG, POCh S.A., Gliwice, Poland, pure), low molecular weight epoxy resin (EPR 0162, Hexion Specialty Chemicals, Inc., Columbus, OH, USA) and the oak wood waste in the form of wood flour, obtained by FHU Parkiety Smolik, during the production of parquet flooring.

### 2.1. Oil Modification and Epoxy Fusion Process

First, epoxy groups in modified soybean oil were opened using ethylene glycol during the five-hour chemical reaction at 110 °C in toluene (POCh S.A., Gliwice, Poland, pure). The molar ratio of glycol to oxirane rings was 1.05. The reaction was performed in the presence of a catalyst in the form of 0.013 moles of sulfuric(VI) acid per 1 mole of oxirane groups in the epoxidized oil. Next, an obtained solution containing hydroxylated soybean oil was (1) washed several times with hot distilled water, (2) distilled under reduced pressure to remove the solvent, (3) characterized, and (4) subjected to the epoxy fusion process with low-molecular-mass epoxy resin in a nitrogen atmosphere. The epoxy fusion process was conducted in the presence of LiCl as a catalyst. The epoxy value of the reacting mixture was monitored for the entire duration of the process to establish an appropriate reaction time. The process was carried out until the assumed content of epoxy groups in the final product was reached. The product was characterized using titration methods (evaluated epoxy and hydroxyl value) and the FT-IR method.

### 2.2. Wood Waste Modification

#### Initial Waste Preparation

The oak wood waste, in the form of wood flour, which was obtained during the production of parquet flooring, was originally characterized by non-uniform particle size. The waste was separated into homogeneous fractions using a laboratory shaker for sieve analysis. The shaker was equipped with a set of seven sieves of various medium dimensions, resulting in separating the wood waste into fractions: >0.2 mm; 0.2–0.16 mm; 0.16–0.1 mm; 0.1–0.076 mm; 0.076–0.056 mm; 0.056–0.04 mm, and <0.04 mm. In further studies, pre-dried at a temperature of 80 °C for 48 h, a fraction with a size <0.04 mm was used. Wood flour was subjected to different types of modifications: mercerization (1), acetylation (2), and MDI-modification (3).

(1) Mercerization: After placing wood flour in a beaker, hot distilled water was added and the suspension was mechanically stirred for 30 min, then the waste was filtered on a Büchner funnel. Mercerization was carried out three times using sodium hydroxide solutions with concentrations of 5 and 10%. Each reaction was carried out for 30 min, then wood waste was neutralized, rinsed with distilled water, and filtered again on a Büchner funnel. The mercerized waste was dried at 80 °C for 48 h. The dried waste was then grounded and re-fractionated using a laboratory shaker for sieve analysis.
(2) Acetylation of wood waste was carried out in a reaction set of the three-necked flask, a mechanical stirrer, a thermometer, and a reflux condenser. Apart from the 100 g of wood waste (previously washed with hot distilled water and mercerized with a 5% solution of NaOH), the flask also contained 4-dimethylaminopyridine (in the amount of 5 wt.% concerning wood flour) and 1000 mL of acetic anhydride. The reaction was carried out for 2.5 h at 120 °C. After the reaction was completed, the mixture was cooled and filtered on a Büchner funnel. The acetylated waste was then mixed with 300 mL of acetone using a mechanical stirrer for 30 min, then the wood flour was filtered and subjected to Soxhlet extraction (2 h for a total of 9 cycles). The extraction mixture consisted of toluene, acetone, and methanol in 4:1:1 volumetric ratios. The acetylated wood waste was also dried at 80 °C for 48 h, then the dried modified waste was grounded and re-fractionated.
(3) MDI-modification: A total of 30 g of wood flour and 100 g MDI-based polyisocyanate (DESMODUR VL, Covestro AG, Warsaw, Poland) were introduced into a 1000 mL beaker, and the suspension was mechanically stirred for 1 h. Then the wood waste was filtered off in a Büchner funnel. The drained WF was transferred into the beaker, 200 mL acetone was added, and the mixture was stirred for another 30 min and filtered on the Büchner funnel again. Mixing with acetone and filtration was performed twice. Then modified wood flour (DWF) was dried for 24 h at 50 °C, grounded, and sieved to obtain a fraction <0.04 mm.

### 2.3. Epoxy Composites with Modified Wood Waste

Unmodified or chemically modified wood waste (respectively in the amount of 2 or 5 wt.%) was mixed for 30 min using a mechanical mixer (700 rpm) with the polyaddition product based on hydroxylated soybean oil and low-molecular-weight epoxy resin (SMEG_EPR). Followed by thorough mixing during an additional 5 min (1000 rpm) weighed amount of hardener (4,4′-methylene diphenyl diisocyanate, MDI) and de-aerating agent (BYK A530, 1 wt.%. concerning the total weight of the composition). Finally, the composition was de-aerated for 3 min at a pressure of 0.8 MPa. Each composition was cured in Teflon molds at room temperature for 24 h, and then at 80 °C for an additional 24 h.

### 2.4. Spectroscopic Measurements

FT-IR PerkinElmer spectrophotometer (SPECTRUM 65 FT-IR, PerkinElmer, Seer Green, UK) with an ATR adapter was used to register changes in chemical structure, which occurred during performed chemical modification of wood waste. All analyses were carried out at room temperature. The small amount of pre-dried waste flour was transferred onto the ZnSe crystal attenuated total reflectance unit (ATR), then evenly pressed with a spatula to cover the crystal with the analyzed sample, and finally, the created layer was pressed down with a screw equipped with an attachment for loose samples to tightly compress the sample of waste flour. Spectra were recorded for a wavenumber in the range of 4000–600 cm^−1^. Within the manuscript, registered spectra are presented using the dependence of transmittance T (%) and wavenumber v (cm^−1^).

### 2.5. Morphological Analysis

A morphological analysis of the obtained materials was conducted using a JEOLJSM-6010LA scanning electron microscope (Tokyo, Japan) at a 5 kV acceleration. In the case of samples of epoxy composites SEM micrographs were recorded of the impact-fractured surfaces of the cured compositions. The approximate dimensions of the samples were 2 × 10 mm. While the analysis of post-modification morphological changes within the natural filler was performed on pre-dried flour particles. In both cases, samples were coated with a thin film of gold.

### 2.6. The Mechanical Properties 

The Mechanical Properties of the prepared composition were tested according to previously described procedures [26] incluxding: PN-EN ISO 527-1:2012 [37], PN-EN ISO 178:2011 [38], PN-EN ISO 604:2006 [39], PN-EN ISO 868:2005 [40] and PN-EN ISO 179-2:2001 [41].

## 3. Results

**Figure 1:**
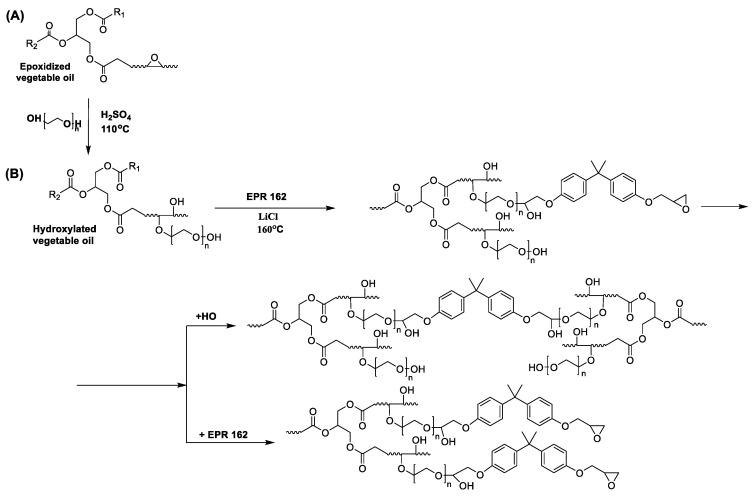
The oxirane ring-opening reaction of epoxidized soybean oil with monoethylene glycol (**A**) followed by the fusion process of hydroxyl soybean oil and low-molecular-weight epoxy resin EPR0162 (**B**).

The high-molecular-weight epoxy resin was synthesized via the epoxy fusion process, conducted via the process carried out following previously performed procedures [21] using modified soybean oil. In the first stage, commercial epoxidized soybean oil was used to obtain the hydroxylated soybean oil (SMEG). SMEG was synthesized during the oxirane ring-opening reaction of epoxidized soybean oil with monoethylene glycol (Figure 1A).

The reaction was carried out with a slight (1.05:1.0) molar excess of glycol to the epoxy groups contained in the ESBO. The process was carried out in the presence of sulfuric acid(VI) as a catalyst at a temperature of 110 °C. During the synthesis, the change in viscosity and the color of the reaction mixture was observed. In the beginning, the epoxidized soybean oil was light yellow (Table 1), and then, as the reaction proceeded, the reaction mixture gradually changed its color to darker until it became brown-orange (Table 1).

**Figure 2:**
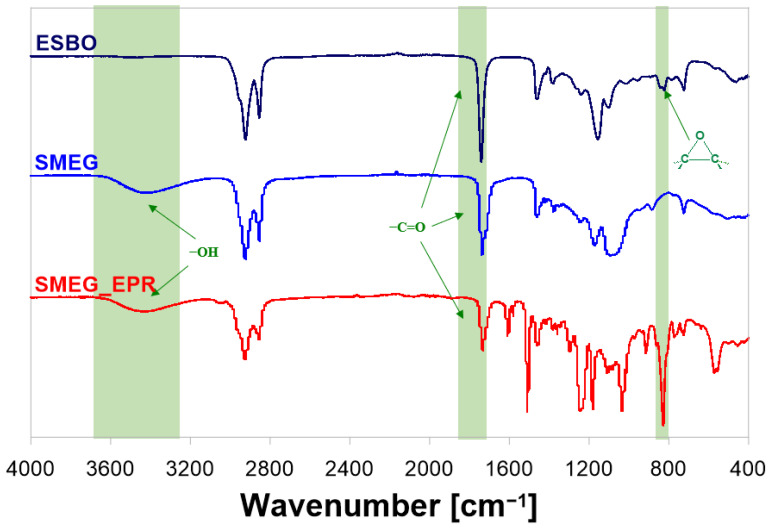
FT-IR spectrum of epoxidized soybean oil, hydroxylated soybean oil, and polyaddition product of hydroxylated soybean oil and low molecular weight epoxy resin.

The color change was accompanied by an increase in viscosity. The reaction was carried out for 4 h. Then the reaction product was cooled to 30 °C, neutralized with calcium carbonate, washed several times with hot distilled water, and subjected to distillation under reduced pressure. The obtained product of the oxirane ring-opening reaction was characterized by a higher viscosity compared to the epoxidized oil. To determine the content of functional groups in SMEG, the oil was subjected to the following titration tests, including the determination of epoxy (EV), acid (AV), and hydroxyl (HV) values (Table 1). Based on the obtained results, it was observed that the epoxidized soybean oil contains a large number of free oxirane groups and relatively few hydroxyl groups. However, after the oxirane ring-opening reaction, no oxirane groups were found. Therefore, it can be concluded that during the performed reaction all rings in epoxidized soybean oil reacted towards obtaining the product characterized by the relatively high amount of hydroxyl groups. Observations were also confirmed by the recorded FT-IR spectrum (Figure 2).

On the spectrum, registered for the epoxidized soybean oil, the presence of characteristic vibrations is observed in the range of wavenumber 840–812 cm^−1^. These signals come from strong, valence vibrations of epoxy groups. Moreover, bands in the range of *v* = 2924–2922 and *v* = 2854–2851 cm^−1^, as a result of the interactions of methyl and methylene groups can be distinguished. The presence of vibrations for *v* = 1740–1737 cm^−1^ is probably related to the strong carbonyl bonds. In turn, the bands of low intensity in the ranges *v* = 1509–1462 cm^−1^, *v* = 1376–1357 cm^−1^, *v* = 1240–1227 cm^−1^, *v* = 1170–1152 cm^−1^ and *v* = 723–721 cm^−1^ are caused by the presence of scissor, deformation and pendulum vibrations of bonds originating from methylene and methyl groups. In the case of the spectrum recorded for the SMEG, the product of the oxirane ring-opening reaction in ESBO, the appearance of a characteristic band in the wavenumber range of 3660–3124 cm^−1^ can be observed, which corresponds to the stretching vibrations of the hydroxyl groups of variable intensity. An important difference between the SMEG versus ESBO spectra is the lack of band *v* = 812–820 cm^−1^ for SMEG, which additionally confirms the conversion of all free epoxy groups in the modified oil.

In the next stage of the research, the polyaddition reaction of hydroxylated soybean oil (SMEG) and low-molecular-weight epoxy resin was carried out (Figure 1B) in the presence of LiCl as a catalyst [21]. The reaction was carried out for 10 h, under an inert gas atmosphere at a temperature of 160 °C. The course of the reaction was controlled by examining the content of free epoxy and hydroxyl groups to obtain the product assumed in advance, the specific content of functional groups, and its processable viscosity. During the SMEG_EPR polyaddition reaction, the initially formed SMEG_EPR molecule can react further with the following hydroxyl groups present in the SMEG and oxirane rings of the EPR resin [19]. An epoxy resin based on hydroxylated soybean oil was in the form of a brown-orange liquid with high viscosity. In the range of *v* = 3660–3112 cm^−1^, a wide derived band from stretching vibrations of the hydroxyl group is observed (Figure 2). It is worth noting that in the case of the SMEG_EPR polyaddition product, the intensity of this band decreased compared to the spectrum recorded for SMEG. This change is also confirmed in the results of the performed determination of the content of -OH groups. Moreover, for the SMEG_EPR polyaddition product, two signals with *v* = 1610 and 1500 cm^−1^ appear, invisible in the spectrum of hydroxylated oil. They correspond to the variable vibrations of the aromatic ring and the asymmetric vibrations of the epoxy groups.

**Figure 3:**
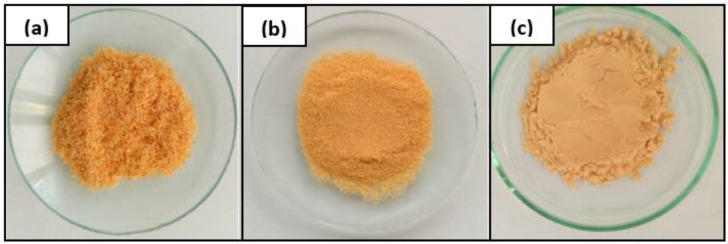
Fractionation of wood flour-particle size of WF: >0.2 mm (**a**), 0.076 mm (**b**) and <0.04 mm (**c**).

In the next stage of the research, the modification of the wood waste intended for filling epoxy compositions based on the obtained polyaddition product was carried out. Wood shavings from the waste from the production of oak parquet were used as the filler. Acquired wood material was characterized by different particle sizes, so it was necessary to refract it to obtain wood flour (WF) with uniform dimensions. Separation of the fractions was performed using sieves, yielding the following fractions:>0.2; 0.2–0.16; 0.16–0.1; 0.1–0.076; 0.076–0.056; 0.056–0.04 and <0.04 mm (Figure 3).

To ensure the best possible degree of homogenization of WF and a polyaddition product, as a filler for epoxy-polyurethane materials, a fraction with dimensions < 0.04 mm was selected. The smallest dimensions of the filler in combination with 30 min of mechanical mixing made it possible to obtain a homogeneous composition without visible agglomeration of the filler in a highly viscous polyaddition product.

**Figure 4:**
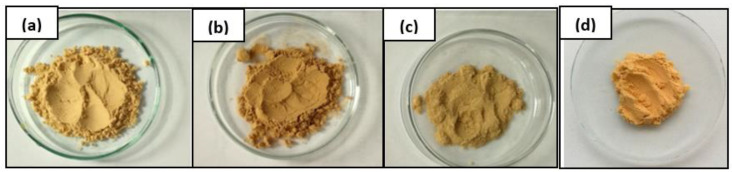
Wood flour: unmodified (**a**), modified using 5% (**b**) and 10% solution of NaOH (**c**), and modified with a 4,4′-diphenylmethane diisocyanate (**d**).

Before further application, the bio-filler was dried at 60 °C for at least 48 h, more specifically until there was no change in weight due to moisture evaporation. Then wood shavings from the waste were modified chemically. Modification of wood flour by alkalization (mercerization) was carried out, using sodium hydroxide solutions of various percentages (5 and 10%). In the first stage, the wood waste was washed with hot distilled water to remove impurities and lead to the initial swelling of the waste, thus increasing access to hydroxyl groups. Then the wood flour was filtered off on the Büchner funnel, however, due to the high degree of swelling, the filtration process was troublesome. In the next stage, wood flour was mixed with a sodium hydroxide solution of the appropriate concentration. During the mixing of WF with the NaOH solution, the color of the wood flour turned brown. Then, after 30 min of alkali exposure, the wood waste was filtered again on a Büchner funnel and treated with a 1M hydrochloric acid solution. After neutralization, the wood waste turned color from dark to light brown (Figure 4a–c).

**Figure 5:**
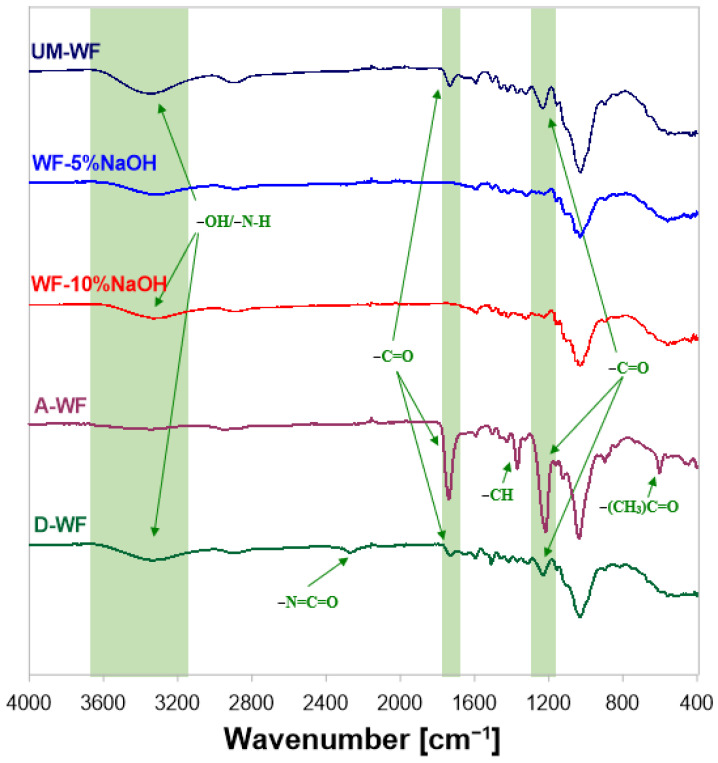
FT-IR spectrum of unmodified and chemically modified wood flour.

The WF was then filtered off and rinsed with distilled water. Next, wood flour, modified by mercerization was dried at the temperature of 80 °C for 48 h. The drying time was selected based on the degree of drying. The WF was dried until there was no further change in the weight of the waste due to moisture evaporation. The wood filler was then ground and again subjected to fractionation to finally obtain WF with a size < 0.04 mm. The change in the color of wood flour could result from the loss of hydroxyl groups and the reduced content of cellulose-derived carbonyl bonds. Another factor determining this phenomenon could be the degradation of lignin, caused by the neutralization of wood flour with hydrochloric acid [42]. To investigate changes in the chemical structure in WF after mercerization, FT-IR analysis was performed. The spectrum shows a clear influence of chemical modification on the chemical structure of the filler. On the FT-IR spectrum (Figure 5) in the range, *v* = 3607–3080 cm^−1^, stretching vibrations of the hydroxyl groups are observed [43], and in this case, it is important to reduce the intensity of this band for WF modified with NaOH solutions.

For modified wood flour with a 5% solution of NaOH, the band corresponding to the -OH groups is characterized by a lower intensity than in the case of WF modified with the 10% solution of NaOH. The transmittance band registered in the wavenumber range *v* = 2980–2822 cm^−1^ is related to the presence of stretching vibrations originating from methyl and methylene groups. In the case of unmodified wood flour, vibrations of low intensity from the C-O-C stretching vibrations can be observed. The signal at *v* ≈ 1728 cm^−1^ is most likely related to the presence of carbonyl stretching bonds in hemicelluloses and lignin. Small signals at the wavenumber *v* ≈ 1590 cm^−1^ can be attributed to the -C=C- skeletal vibrations in the aromatic rings of the lignin structure. On the spectrum corresponding to unmodified wood flour, the band in the range *v* = 1297–1188 cm^−1^ most likely corresponds to the -C-O stretching vibration from lignin. The intense band in the wavenumber range *v* = 1178–883 cm^−1^ is the result of the interaction of bonds present in lignin and cellulose molecules. Its intensity may be influenced by C-O-C stretching vibrations in pyranose rings and -C-O vibrations in aliphatic groups [44,45].

In the next stage, wood flour was modified by acetylation. Acetylation of wood material allows for the elimination of hydroxyl groups contained in its structure [46]. Accordingly, better adhesion between the WF and the polymer matrix can be achieved. The acetylation of the wood waste was carried out with acetic anhydride in the presence of 4-dimethylaminopyridine as a catalyst. The reaction was carried out at 120 °C for 2.5 h. After the completion of the reaction, the reaction mixture was cooled and filtered on a Büchner funnel. The resulting waste was washed with acetone and subjected to Soxhlet extraction. Extraction was performed in the presence of a toluene-acetone-methanol mixture (4:1:1 by volume) for 2 h. The obtained modified wood waste was dried at the temperature of 80 °C for 48 h. Then, the modified wood flour was ground and again sieved through sieves to finally obtain the acetylated wood flour with a size < 0.04 mm. On the FT-IR spectrum (Figure 5), the lack of a band characteristic for hydroxyl groups in the range of *v* = 3600–3000 cm^−1^ and the appearance of signals at *v* = 600 cm^−1^ indicates the replacement of hydroxyl groups with acetyl groups derived from the anhydride. The signals in the wavenumber range *v* = 2980–2822 cm^−1^ can be attributed to the tensile forces of the -CH groups. In turn, the signal at *v* ≈ 1728 cm^−1^ results from the occurrence of the derived valence vibrations from carbonyl bonds in the structure of hemicellulose and lignin and is particularly visible in the case of the spectrum recorded for acetylated wood flour. The vibrations of relatively small intensity at *v* = 1595 cm^−1^ may result from the interactions of the skeletal groups -C=C- present in aromatic rings. In addition, in the spectrum of acetylated wood flour (A-WF), there is a band at the wavenumber *v* ≈ 1365 cm^−1^, which may be related to deformation -CH vibrations in cellulose and hemicelluloses. The change in vibration intensity in this area may indicate the overlapping of bands corresponding to -CH groups with a signal characteristic for the acetyl group (stretching -C-CH_3_ vibrations and bending -C-H vibrations), which may also occur in this range [47]. The band in the wavenumber range 1296–1180 cm^−1^, both for unmodified and modified WF, is the result of the vibration of stretching -C-O bonds in the lignin molecule. As in the case of wood waste subjected to mercerization, the band characterized by the relatively high intensity in the range of 1178–860 cm^−1^ is the effect of -C-O stretching vibrations in lignin and cellulose particles. The signal at *v* ≈ 600 cm^−1^, recorded for the A-WF, corresponds to the deformation vibrations of the acetyl groups, as a result of the performed modification [44,48].

Additionally, the modification of the wood flour was also carried out with the use of 4,4′-diphenylmethane diisocyanate. In the first stage of modification, the wood material was mixed with the polyisocyanate using a mechanical mixer. The wood flour was then drained on a Büchner funnel and washed twice with acetone. After the modification was completed, the WF was filtered off and dried at 80 °C for 48 h. During the modification process using diisocyanate no difficulties, such as those observed in previously performed modifications, were noted. The modified wood waste was characterized by a much lower degree of swelling than mercerized WF. Similar to previous modifications, after completing the modification process, the diisocyanate-modified wood flour (DWF) was grounded and fractionated using sieves with a size < 0.04 mm. The obtained filler was characterized by an intense yellow color (Figure 4d). On the FT-IR spectrum (Figure 5) of wood flour, before and after modification, a signal with lower intensity in the range of 3642–2999 cm^−1^, attributed to the presence of hydroxyl groups in the tested sample, was registered for MDI-modified wood flour. Additionally, a significant observed difference was in the appearance of the signal at the wavenumber at *v* = 2271 cm^−1^. Registered vibrations are the result of the interaction of free-NCO groups [49].

**Figure 6:**
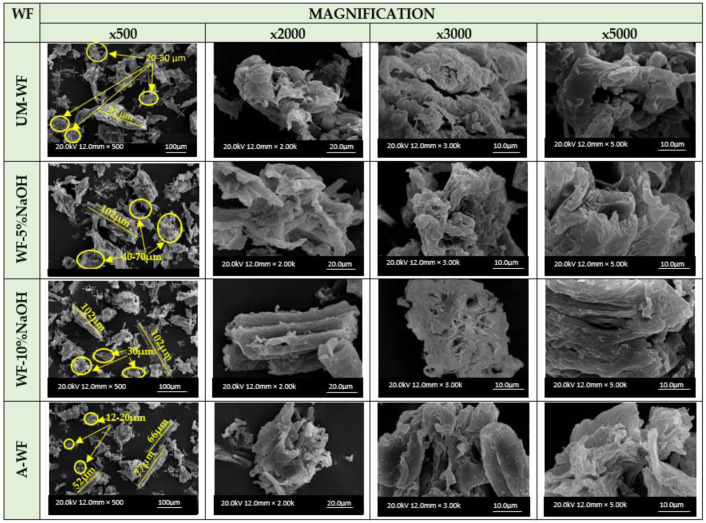
SEM micrographs of unmodified and modified wood flour.

As mentioned in the introduction, on the one hand, the main emphasis in the presented research was placed on the use of bio-waste in vegetable-based epoxy composites and on examining the effect of introducing bio-waste into the polymer matrix. However, on the other hand, apart from the analysis of changes in the chemical structure using the FT-IR method, it seems also interesting to pay attention to changes in the morphology of waste wood flour subjected to chemical modifications. Therefore, selected samples of wood filler were subjected to SEM analysis (Figure 6).

As seen in the presented microphotographs, the performed modifications had a major influence on the size of wood particles. In general, within unmodified wood samples, most of the wood particles are about 23–39 µm, there are also noticeable larger particles such as the one of about 91 µm. In turn, in the case of mercerized wood flour, the effect of alkali treatment might be seen e.g., in the mean particle sizes. In the case of wood flour subjected to mercerization using a 5% solution of NaOH, obtained particles are mostly about 46–68 µm, with some about 102 µm. Similarly, in the case of treatment with a 10% solution of alkali, larger particles about 102 µm are present, however, most particles are about 30 µm. The swelling reaction of wood filler was mentioned before with the observations made during the conducting of the modification process. At that time, occurred swelling significantly influenced the filtration process of wet filler. According to the research described in the literature, the swelling reaction of wood filler is correlated with the cellulose material response to alkali treatment. During mercerization, the natural crystalline structure of cellulose relaxes [50]. This is a very important phenomenon for performing further chemical treatments of wood filler considering the structural construction of cellulose. It is well-known that lignocellulosic materials are composed of crystalline and amorphous regions, which differ in behavior from chemical reagent treatments. While the amorphous regions easily absorb chemicals, the crystalline regions are difficult for chemical penetration [51]. However, during the mercerization process, the decrystallization of cellulose occurs, thanks to which the potential further treatment with different chemicals is more effective. In addition to differences in the dimensions of wood particles, that occurred during WF modification, the presented samples also exhibit disparateness in the morphological structure. The surface of unmodified WF, presented on SEM microphotographs, is uneven with visible irregular shreds that have been created during wood processing by sanding. Here, it is worth mentioning that the chemically untreated wood surface is also covered with pectin, lignin, hemicellulose, waxy substances, and impurities, which might be removed during the mercerization process [52]. In turn, the surface of wood flour after the treatment with an alkaline solution became even more rough and uneven with a larger number of irregular shreds. However, these irregular shreds are not visible on the microphotographs presenting acetylated wood filler.

**Figure 7:**
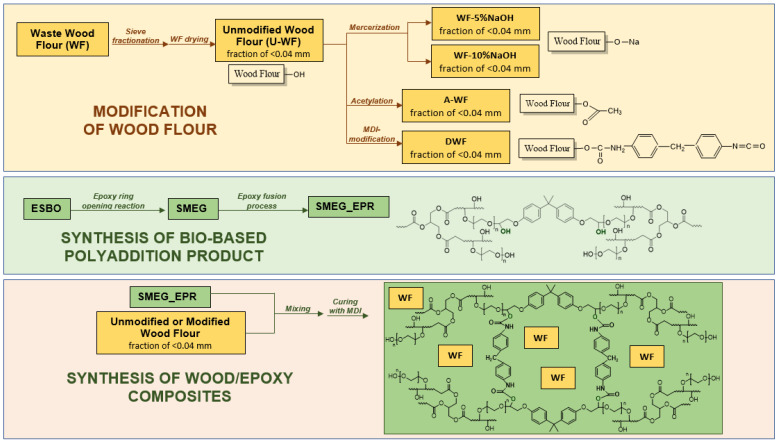
Scheme of the synthesis of wood/epoxy composites based on high molecular weight epoxies from hydroxylated soybean oil and bisphenol A-based epoxy resin filled with waste wood flour.

Finally, in the next stage of research, epoxy composites based on SMEG_EPR polyaddition products were obtained (Figure 7). Unmodified wood flour, wood flour modified by mercerization, acetylation, and isocyanate were used as the dispersed phase. First, wood flour was introduced to the SMEG_EPR polyaddition product in an amount of 2 or 5 wt.% based on the total weight of the prepared composition. Then, the whole mixture was stirred mechanically for 30 min. BYK A530 defoamer (1 wt.% based on the total weight of the prepared composition) was added successively to facilitate the removal of air bubbles formed during the mixing process. Subsequently, 4,4′-diphenylmethane diisocyanate (MDI), used as a hardener, was added. The amount of curing agent, necessary to cross-link the epoxy materials, was calculated taking into account the content of hydroxyl groups in the polyaddition product. Each composition was poured into Teflon molds and hardened for 48 h at room temperature, then the specimens for mechanical tests were taken out of the molds and subjected to crosslinking at the temperature of 80 °C for the following 24 h.

Attempts were made to create epoxy-polyurethane compositions filled with all prepared wood flour samples, however, the material obtained using DWF was characterized by an unsatisfactory appearance. Unfortunately, during the curing process, numerous air bubbles were formed resulting in obtaining the product of almost doubled dimensions. This could be due to the faster reaction of the -NCO groups, both from the modified filler and the hardener, with moisture from the air, than with the appropriate, derived from the polyaddition product, hydroxyl groups. Therefore, no further tests were carried out on composites filled with MDI-modified wood flour.

**Figure 8:**
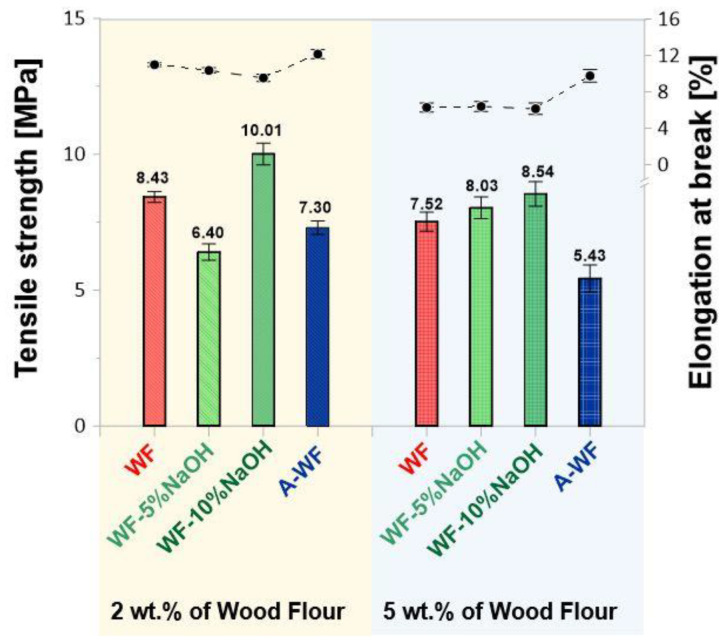
Tensile strength and elongation at break of compositions based on SMEG_EPR filled with wood flour.

Based on the mechanical tests, it was observed that the addition of a natural filler caused a reduction in the mechanical strength of the composites as compared to the reference samples–the one without the filler. The reference sample, SMEG _EPR composition, was characterized by a tensile strength of 21.36 MPa, with a relative elongation at a break of 3.49% and a modulus of elasticity of 460.33 MPa (Figure 8).

The introduction of wood flour caused an almost threefold reduction in tensile strength, with a simultaneous increase in the value of the relative elongation at break, in the reference with unfilled composites based on SMEG_EPR. The registered value of tensile strength for SMEG_EPR_2%WF composition was 8.43 MPa, while a further increase in the amount of the wood filler up to 5 wt.% resulted in the additional reduction of this value up to 7.52 MPa. Generally, comparing all obtained epoxy compositions containing wood flour, better mechanical results were recorded for samples containing the chemically modified natural filler. However, the obtained values were unfortunately still lower than those received for unfilled reference composition. Samples containing 2 wt.% of wood flour modified with a 5% sodium hydroxide solution (SMEG_EPR_2%WF-5%NaOH) were characterized by the tensile strength of 6.40 MPa, and it was the lowest value of all compositions containing wood flour modified by mercerization. For the composition containing 5 wt.% of WF-5%NaOH, the tensile strength was found to be 8.03 MPa. The introduction of the natural filler modified with a 10% solution of NaOH resulted in further improvement of tensile strength concerning composites with WF modified with a 5% NaOH solution. Nevertheless, these values were still lower than the reference sample. For both mercerized fillers (wood flour modified with 5 and 10% NaOH solutions), along with an increase in fillers’ weight fraction, the value of the relative elongation at break decreased. In turn, compositions containing wood flour modified by acetylation, compared with mercerized wood flour, exhibit lower tensile values in general. A composition containing 2 wt.% of A-WF was characterized by the tensile strength of 7.31 MPa, with relatively, the highest of all values of elongation at break. Simultaneously, with the increase in the weight fraction of acetylated wood flour the tensile strength decreased (5.43 MPa for composite containing 5 wt.% A-WF).

**Figure 9:**
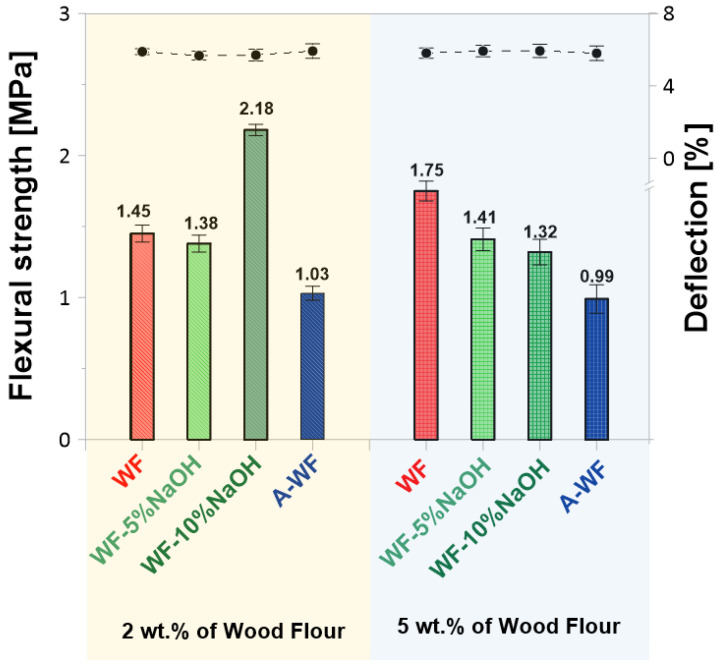
Flexural strength and deflection of compositions based on SMEG_EPR filled with wood flour.

A similar and comparable tensile strength relationship was also recorded during the performed bending strength tests (Figure 9).

The values obtained for epoxy/wood composites were 2–3 times lower compared to the sample without the natural filler. Wherein, among all wood-filled compositions, SMEG_EPR_2%WF-10%NaOH exhibits the most outstanding bending properties (flexural strength 2.18 MPa, deflection of 5.91% compared with 1.46 MPa and 5.88% for SMEG_EPR_2%WF, respectively).

**Figure 10:**
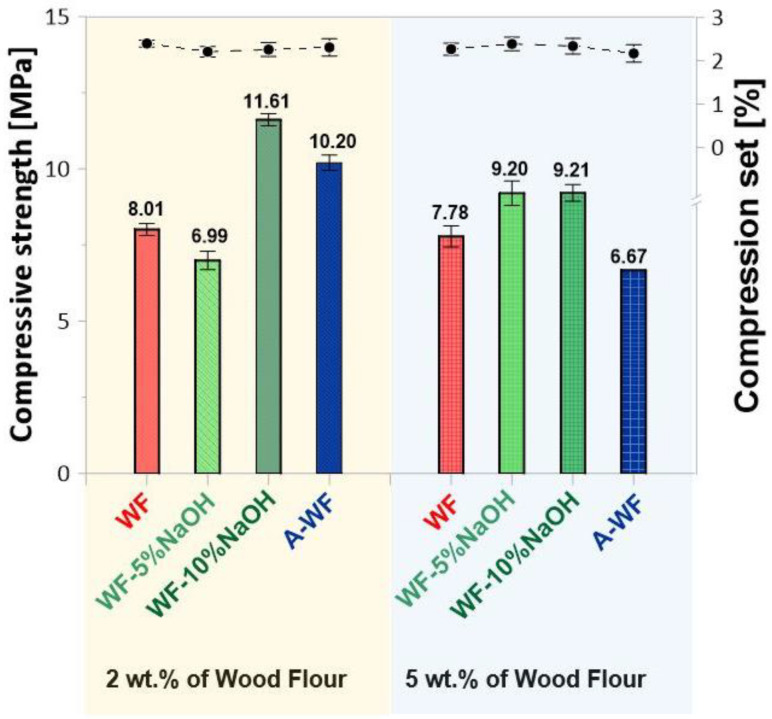
Compressive strength and compression set of compositions based on SMEG_EPR filled with wood flour.

Performed chemical modification of wood flour seems to have the greatest effect on the compressive strength properties of tested composite samples (Figure 10).

Nevertheless, all of them exhibit lower compressive strength values compared to the samples without the wood filler. However, when comparing results within the group of all epoxy/wood composites, recorded compressive properties for chemically modified samples present significant improvement. Appropriately, 11.61 MPa for SMEG_EPR_2%WF-10%NaOH, 10.20 MPa for SMEG_EPR_2%A-WF and 8.01 MPa for SMEG_EPR_2%WF, respectively. Simultaneously, as in the case of previously discussed mechanical properties, the tensile and flexural strength samples containing 2 wt.% of WF modified with a 10% solution of NaOH exhibit the highest values. However, for all composites containing 5 wt.% of wood flour modified with NaOH solution–the increase in weight fraction of the filler, improved the mechanical properties, while for composites with 10 wt.% of WF-10%NaOH–the increase of weight fraction of the modified wood affected the deterioration of recorded compressive properties.

All tested compositions were characterized by high values of Shore A hardness (91–93°Sh), within the highest values for SMEG_EPR and SMEG_EPR-5%WF (Table 2).

Also, in the case of impact strength, the greatest value was characteristic for the composition, which did not contain the natural filler (14.06 kJ/m^2^), while the lowest value was obtained for the composition SMEG_EPR_2%WF-5%NaOH and SMEG_EPR_5%A-WF, respectively 4.25 kJ/m^2^ and 3.89 kJ/m^2^.

**Figure 11:**
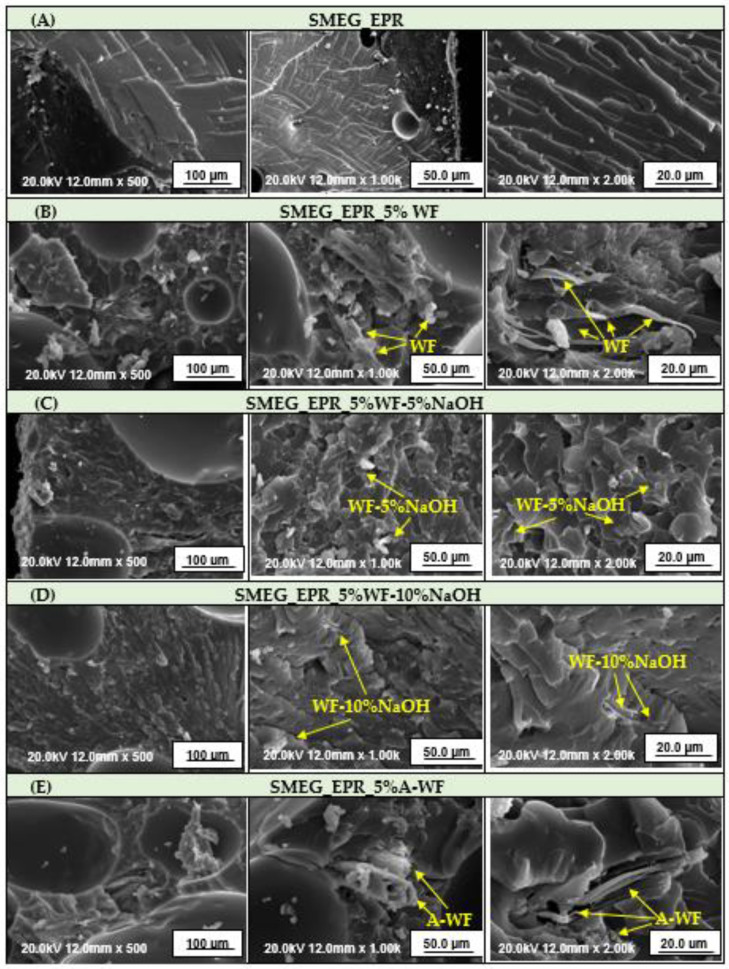
SEM micrographs of the impact fracture surface of the crosslinked sample of (**A**) reference SMEG_EPR composition (**B**) SMEG_EPR composition filled with 5 wt.% of unmodified wood flour, (**C**) SMEG_EPR composition filled with 5 wt.% of wood flour mercerized using a 5% solution of NaOH, (**D**) SMEG_EPR composition filled with 5 wt.% of wood flour mercerized using a 10% solution of NaOH, and (**E**) SMEG_EPR composition filled with 5 wt.% of acetylated wood flour.

Samples of selected compositions were also subjected to the morphology analysis, conducted by the SEM method (Figure 11). Performed analysis reveals that the epoxy-polyurethane compositions based on modified soybean oil do not show brittle cracks, which are characteristic of conventional resins. There is also no phase separation characteristic, for example, for cross-linked epoxy materials with the addition of natural resources as reactive diluents [53]. In the case of mentioned materials, the oil microdomains are observed evenly dispersed throughout the sample volume [54]. On microphotographs presenting composite epoxy-polyurethane samples containing the unmodified wood filler-cured SMEG_EPR composition filled with 5 wt.% of unmodified wood flour agglomerates unevenly distributed throughout the composition were found. The presence of smaller and larger agglomerates could explain poor interfacial affinity and lower values of mechanical properties presented by wood flour/epoxy composites versus unfilled epoxy-polyurethane material. Performed mercerization and acetylation of wood flour, as discussed before allows for the elimination of hydroxyl groups contained in its structure, which potentially leads to an increase in the compatibility between epoxy-polyurethane matrix and wood flour. However, as seen on microphotographs, the interface between individuals is still noticeable. Microphotographs of epoxy-polyurethane compositions and wood flour indicate the fiber pullout from the polymer matrix. However, in the case of modified wood flour, separating wood fibers are slightly more coated by the polymer compared to the one containing unmodified wood flour. On the other hand, even though, as seen on the presented microphotographs, the chemical treatment via the acetylation increased the compatibility between epoxy-polyurethane matrix and wood flour, registered values of tensile and flexural strength were insignificantly lower than those obtained for samples containing unmodified wood flour. However, the effect of modification as better compatibility of filler and polymer matrix is visible in the case of compressive strength. Composition SMEG_EPR_2%WF-10%NaOH is characterized by a 3.51 MPa and SMEG_EPR_2%A-WF by 2.19 MPa higher value of compressive strength than samples containing unmodified wood flour.

## 4. Conclusions

In the presented research commercial epoxidized soybean oil was modified by opening the oxirane rings to give a hydroxylated derivative of vegetable oil. Next hydroxylated soybean oil was used in a polyaddition reaction to obtain epoxy resins. The polyaddition products based on hydroxylated soybean oil contain epoxy and hydroxyl groups, which allows cross-linking of the composition using conventional hardeners suitable for epoxy resins, as well as polyisocyanates. The main purpose of undertaken research was an attempt at functional management of waste wood flour towards a ‘greener’ approach to the synthesis of bio-based epoxy resins. To reduce the hydrophilicity of the wood filler, a chemical modification was performed. It was found that both, mercerization and alkalization, lead to a reduction of the content of hydroxyl groups and compounds such as lignin and hemicellulose within the structure of wood. Additionally, bonds, which are formed during the acetylation in the structure of the filler have a positive effect on the interaction of the polymer matrix-wood flour. Comparing two performed methods of modification of wood flour, it was found that the modification of wood waste by acetylation allows for the hydroxyl groups removal to a greater extent than in the case of mercerization.

As a result of the performed synthesis, which included (1) the synthesis of SMEG_EPR polyaddition product, (2) the introduction of WF followed by its (3) curing with diisocyanate, obtained wood/polymer composites containing about 40% of raw materials of natural origin. Moreover, it was found that on one hand, compared with the reference sample, the introduction of the oak wood waste in the form of unmodified or chemically modified wood flour to bio-based high molecular weight epoxy resins of hydroxylated soybean oil and a low-molecular-weight epoxy resin resulted in deterioration of tensile and flexural strength. However, on the other side, performed a chemical modification of wood flour had the greatest effect on the compressive strength properties of tested composite samples.

## Figures and Tables

**Table 1:** Characteristics of modified soybean oil and epoxy fusion product SMEG_EPR.

Product	Color of the Sample	EV [mol/100 g]	AV [mg KOH/g]	HV [mg KOH/g]
ESBO	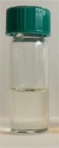	0.363	0.260	41
SMEG	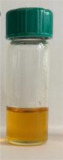	0.000	0.709	253
SMEG_EPR	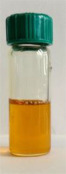	0.208	0.318	152

**Table 2:** Mechanical properties of compositions based on SMEG_EPR filled with wood flour.

Mechanical Properties	Tested Epoxy-Polyurethane Compositions Based on SMEG_EPR							
	Unmodified Wood Flour		Modified Wood Flour					
			5% NaOH		10% NaOH		Acetylation	
	2% WF	5% WF	2% WF	5% WF	2% WF	5% WF	2% WF	5% WF
Modulus of elasticity [MPa]	229.38 ± 8.60	197.42 ± 30.57	117.23 ± 9.28	203.30 ± 28.03	278.00 ± 14.32	220.03 ± 30.50	186.94 ± 15.61	144.50 ± 18.28
Elasticity flexural modulus [MPa]	137.25 ± 15.92	185.00 ± 5.29	136.75 ± 21.90	126.20 ± 30.26	245.20 ± 49.84	112.33 ± 4.73	85.00 ± 13.29	103.75 ± 8.42
Shore Hardness [Sh°A]	90.80 ± 3.99	93.90 ± 2.13	91.50 ± 3.24	90.50 ± 4.28	92.50 ± 3.31	90.60 ± 3.92	90.50 ± 3.31	91.20 ± 1.93
Impact toughness [kJ/m^2^]	6.16 ± 1.23	4.37 ± 0.99	4.25 ± 0.59	5.40 ± 0.72	10.55 ± 0.73	7.38 ± 1.16	5.39 ± 1.07	3.89 ± 0.33

## Data Availability

Data available on request.
